# Uterine Arteriovenous Malformation Presenting with Amenorrhea—A Rare Case Report and Literature Review

**DOI:** 10.3390/reports8030161

**Published:** 2025-09-01

**Authors:** Hanna Gruber, Dirk O. Bauerschlag, Chie-Hee Cho, Aimée B. Herzog, Ibrahim Alkatout, Melanie Schubert

**Affiliations:** 1 Department of Gynecology and Reproductive Medicine, University Hospital Jena, 07745 Jena, Germany; 2 Department of Diagnostic and Interventional Radiology, University Hospital Jena, 07745 Jena, Germany; 3 Department of Gynecology and Obstetrics, University Hospitals Schleswig-Holstein, Campus Kiel, 24105 Kiel, Germany

**Keywords:** uterine arteriovenous malformation, abnormal vaginal bleeding, uterine artery embolization, doppler ultrasonography, case report

## Abstract

**Background and Clinical Significance:** Uterine arteriovenous malformation (AVM) is a rare but potentially life-threatening condition due to the risk of severe acute hemorrhage. Unlike AVMs in other anatomical locations, which are predominantly congenital, uterine AVMs are often acquired, typically developing after uterine procedures such as dilatation and curettage (D&C), cesarean section, or pregnancy-related events. Women commonly present with abnormal bleeding disorders. **Case Presentation:** We are presenting the case of a 41-year-old Caucasian woman with secondary amenorrhea three months after vacuum curettage in the 7th week of pregnancy. Based on her clinical history and the findings on transvaginal sonography (TVS), uterine AVM was highly suspected. Contrast-enhanced magnetic resonance imaging (MRI) confirmed the diagnosis. The patient underwent successful embolization of the left uterine artery. Follow-up examinations demonstrated complete resolution of the vascular malformation, and regular menstrual cycles resumed during her recovery. With the increasing frequency of uterine surgical interventions, the incidence of uterine AVMs is also expected to rise. The clinical impact is significant when fertility preservation and family planning are still ongoing. To the best of our knowledge, this is the first reported case in which amenorrhea is the primary presenting symptom of a uterine AVM. **Conclusions:** Given the high risk of life-threatening hemorrhage associated with undetected or incorrectly treated AVMs, their presence must always be carefully ruled out in case of bleeding disorders after pregnancy or uterine surgery. Accurate diagnosis prior to any further intrauterine interventions, such as curettage, is crucial to prevent severe complications and ensure appropriate management. In order to avoid life-threatening complications, the possibility of uterine AVM should be considered in the differential diagnosis even in the presence of amenorrhea. The proposed diagnosis and treatment algorithm for uterine AVMs can help avoid misdiagnosis.

## 1. Introduction and Clinical Significance

Arteriovenous malformation (AVM) is often a congenital vascular anomaly characterized by direct flow between arteries and veins, bypassing the capillary bed. It is most commonly reported in vessels of the brain and central nervous system vasculature, as well as in the pulmonary and muscular systems. Incidence rates are reported between 0.69 and 1.42 per 100,000 children, with no gender-based differences observed [1,2]. AVMs exhibit dynamic variability in both size and clinical presentation, ranging from asymptomatic cases to those with non-specific symptoms as pain, hypoxia, or paralysis. In severe cases, AVMs may present with acute rupture, leading to life-threatening hemorrhage depending on their anatomical location [3].

In gynecology, uterine AVMs are rarely reported and, when described, typically involve a direct connection of a branch of a uterine artery and the myometrial venous plexus. Their etiology may be congenital or acquired. Acquired uterine AVMs of the uterus are most commonly the result of trauma, often due to surgical intervention, and are characterized by a singular direct arteriovenous communication between the branches of the uterine artery and the myometrial venous plexus. In contrast, congenital AVMs arise from aberrant differentiation of the primitive capillary plexus during fetal angiogenesis. These lesions tend to be more complex, featuring multiple arteriovenous connections with an intervening nidus and frequently by the involvement of extrauterine vessels [4].

The exact incidence of uterine AVM remains uncertain but is estimated to be approximately 0.63% following delivery or abortion [5]. With the increase in uterine interventions, such as cesarean section or curettage, this number is expected to rise. AVMs predominantly occur in women of typical reproductive age, with a peak incidence around 30 years [6,7]. While congenital AVMs exist, the majority of uterine AVMs are acquired, often resulting from surgical interventions such as cesarean section, curettage, or myomectomy, as well as pregnancy itself. Additionally, etiological factors include infection, gestational trophoblastic disease (GTD), gynecologic malignancies, and prior exposure to diethylstilbestrol [7,8]. The exact pathophysiologic mechanism remains uncertain. However, it is known that the presence of AVM is highest in patients after childbirth or abortion. The reason for this is the hyperestrogenic state, which leads to abnormal angiogenesis and vascularity [4]. Uterine AVMs may be asymptomatic or present with non-specific symptoms such as irregular menstrual bleeding, dyspareunia, lower abdominal pain, or anemia. In severe cases, they pose a significant risk of life-threatening hemorrhage [9]. AVMs have been reported to account for approximately 1–2% of all cases of genital and intraperitoneal bleeding in the literature [4]. When presented with uterine bleeding, important differential diagnoses of uterine AVMs are gestational trophoblastic disease (GTD), retained placental tissue, adenomyosis uteri, and other neoplasia of the endometrium and myometrium, as discussed below.

Digital Substraction Angiography (DSA) with contrast agent remains the gold standard for diagnosing AVMs as well as the gold standard for treating AVMs in combination with embolization. However, noninvasive imaging modalities such as transvaginal sonography (TVS) with color Doppler and magnetic resonance imaging (MRI) are highly effective diagnostic tools that are more widely accessible [9].

Various treatment options are available, ranging from expectant management and medical therapy to interventional and surgical approaches, including uni- or bilateral uterine artery ligation and hysterectomy. Medical therapies may involve the use of uterotonic agents such as progestins, hormonal suppression with gonadotropin-releasing hormone agonists (GnRH-a), or methotrexate. While these options have been explored in the management of uterine AVMs, there is currently no consensus regarding their efficacy, optimal regimen, or role in treatment [5]. In recent years, angiographic arterial embolization has emerged as the preferred treatment modality and gold standard due to its minimally invasive nature, low complication rate, and ability to preserve the uterus, and thereby maintain fertility potential [9].

To the best of our knowledge, this case report is the first description of a uterine AVM presenting primarily with secondary amenorrhea following uterine surgery, rather than with the more commonly described symptoms of irregular or heavy menstrual bleeding. This highlights the importance of considering AVM in the differential diagnosis of any menstrual irregularity, particularly in young women with a history of uterine surgical intervention. Careful anamnesis and thorough ultrasound evaluation are essential for early detection and appropriate management.

## 2. Case Presentation

The 41-year-old woman, gravida VII, para III, was referred to our department with a high suspicion of a trophoblastic tumor due to a high intrauterine vascularity in the ultrasound and persistent detection of ß-human chorionic gonadotropin (ß-hCG) 3 months after vacuum curettage for termination of pregnancy at 7 weeks. Her obstetric history included three spontaneous vaginal deliveries, two medically induced abortions, and one abortion, and, more recently, a termination of pregnancy treated by vacuum curettage at 7 weeks of pregnancy. During her last delivery 8 years ago, placental retention occurred with grade III atony, necessitating manual placental removal followed by instrumental curettage. Family planning was considered complete. Her most recent pregnancy was unintended and managed by vacuum curettage, given the history of grade III atony in her previous spontaneous delivery. The surgical vacuum curettage was performed without complications at an external facility three months prior to presentation. Since the procedure, she had experienced secondary amenorrhea and persistently elevated ß-hCG levels, though she reported no abdominal pain or other associated symptoms at the time of presentation.

Apart from the aforementioned curettages, she had no history of prior uterine or abdominal interventions. Her regular medication included venlafaxine and lithium for the treatment of diagnosed depression, as well as a combination bronchodilator (Foster^®^) for allergic bronchial asthma.

### 2.1. Clinical Findings and Diagnostic Assessment

**Figure 1:**
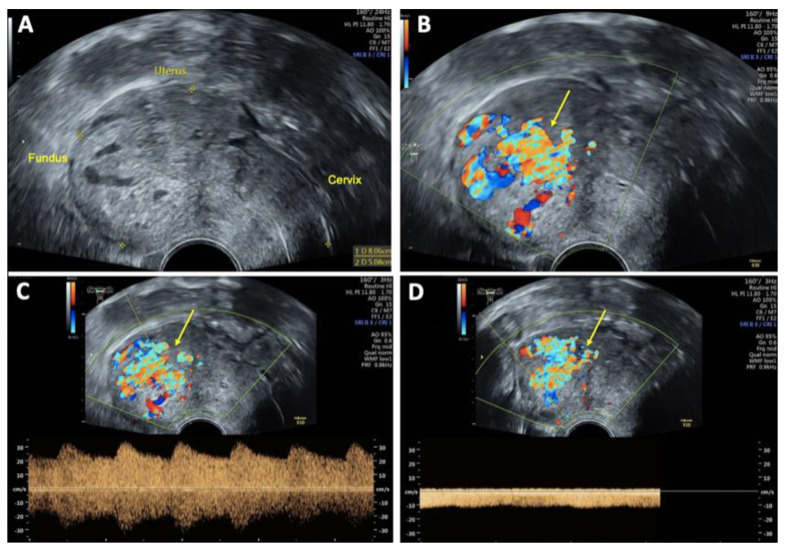
Transvaginal sonography (TVS) Imaging of uterine arteriovenous malformation (AVM): (**A**) 2D TVS demonstrating a retroflected, enlarged uterus measuring 81 × 51 mm with an inhomogeneous structure in the uterine cavity and fundus; (**B**) 2D TVS with color Doppler revealing a vascular tangle suggestive of an AVM (arrow); (**C**) 2D TVS with color Doppler illustrating arterial blood flow within the lesion; (**D**) 2D TVS with color Doppler depicting venous blood flow in the affected area.

**Figure 2:**
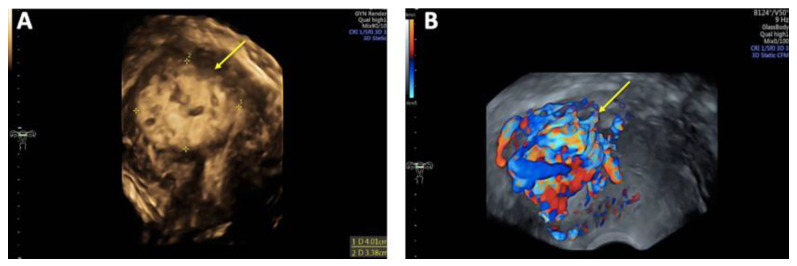
Three-dimensional transvaginal sonography (3D TVS) Imaging of uterine arteriovenous malformation (AVM): (**A**) 3D TVS revealing a 40 × 34 mm inhomogeneous mass (arrow) within the myometrium and uterine cavity; (**B**) 3D TVS color Doppler demonstrating a hypervascular lesion with turbulent blood flow characteristic of a uterine AVM (arrow).

On vaginal examination, no abnormalities or active bleeding were observed. Transvaginal ultrasound revealed a retroflexed uterus measuring 81 × 51 mm, with a suspicious, irregular, hypoechoic, hypervascular lesion measuring 40 × 34 mm on the posterolateral wall of the uterus. This lesion appeared as a prominent vascular tangle extending toward the uterine cavity. Color Doppler sonography demonstrated high-velocity blood flow within the lesion, with a peak systolic velocity (PSV) of 0.3 m/s and a low-resistance pattern, indicated by a resistance index (RI) of 0.43. Distinct arterial and venous blood flow patterns were clearly visualized within the suspicious area (Figure 1 and Figure 2). Both ovaries exhibited normal physiological morphology, appearance, size, and follicle count without abnormalities.

Laboratory analysis in our clinic revealed a ß-hCG level of 13.0 mIU/mL and alpha-fetoprotein (AFP) of <2.8 ng/mL with no other significantly altered laboratory parameters. Based on these findings, the differential diagnoses included placental remnants, trophoblastic tumor, and, most likely, uterine AVM.

**Figure 3:**
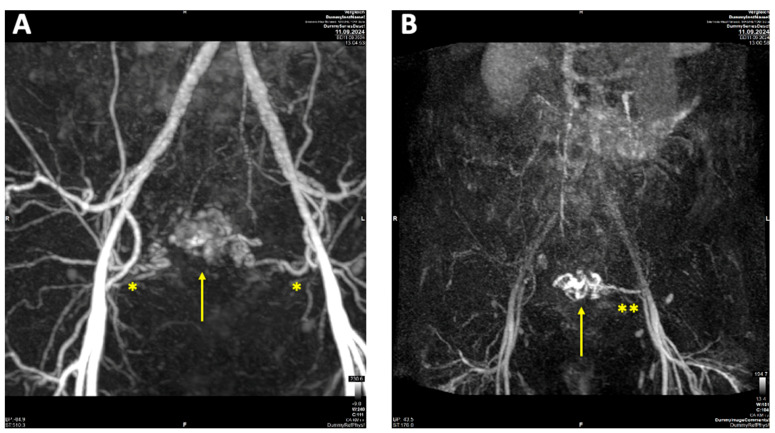
Contrast-enhanced magnetic resonance imaging (MRI) of uterine arteriovenous malformation (AVM): (**A**) MRI angiography with Maximum intensity Projection (MIP) arterial phase demonstrating feeding arteries originating from both uterine arteries (*), with a predominant supply from the left uterine artery; (**B**) MRI MIP venous phase illustrating venous drainage primarily via the left uterine vein (**).

A contrast-enhanced MRI angiography scan was performed to further confirm the diagnosis. Following administration of 6 mL Gadovist^®^, MRI revealed a 45 × 32 mm AVM involving both the myometrium and endometrium of the left ventrolateral uterine wall, extending to the fundus. The AVM was supplied by feeding arteries from both uterine arteries, with a predominant contribution from the left uterine artery. Venous drainage was observed via the left uterine vein, further supporting the diagnosis (Figure 3).

### 2.2. Therapeutic Intervention

After being thoroughly informed about her condition, the patient was counseled on the available treatment options, including expectant management, medical therapy by progestin, GnRH-a or methotrexate, hysteroscopy, uterine artery embolization (UAE) for uterine preservation, and hysterectomy. The patient had completed her family planning and wanted maximum safety with rapid and effective treatment of the AVM while maintaining her uterus. Due to the extent of the AVM, the clear confirmation of the diagnosis by Digital Substraction Angiography, and, after weighing the risks and benefits of hysteroscopy with possible provocation of massive uterine bleeding, we decided against hysteroscopy. After careful consideration of the associated risks and benefits of the other therapy options, the patient opted for uterine embolization. The ß-hCG level dropped to 7 mIU/mL 8 days before the procedure, and, one day before the procedure, it was almost under threshold (2 mIU/mL), which supported our most likely diagnosis of uterine AVM.

**Figure 4:**
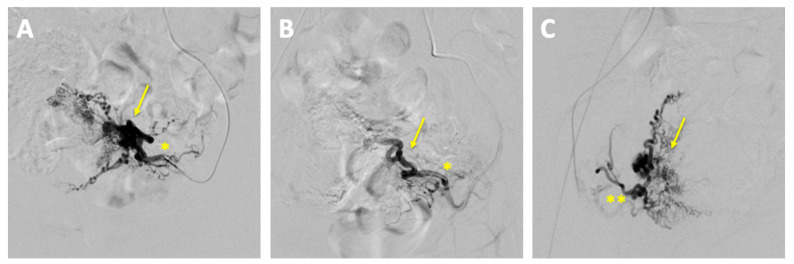
Pre- and post-embolization angiography of uterine arteriovenous malformation (AVM). (**A**) Pre-embolization angiography: superselective catheterization of the left uterine artery (*) demonstrating the vascular supply to the AVM (arrow); (**B**) post-embolization angiography (left uterine artery *): contrast injection via the left uterine artery showing successful embolization with no residual filling of the AVM (arrow) from the left side; (**C**) post-embolization angiography (right uterine artery): contrast injection via the right uterine artery (**) confirming no residual AVM filling from the right side.

The embolization was performed by the department of interventional radiology. The left uterine artery was successfully embolized with 15 mL of Embozene 900 µm, achieving complete stasis of the blood flow. Post-procedural angiography demonstrated a significant reduction in vascularity with markedly slowed blood flow (Figure 4).

Due to the presence of a strong collateral vessel supplying the right ovary and the absence of a detectable feeding vessel from the right uterine artery, embolization of the right uterine artery was not performed in order to preserve ovarian function.

### 2.3. Follow-Up and Outcomes

**Figure 5:**
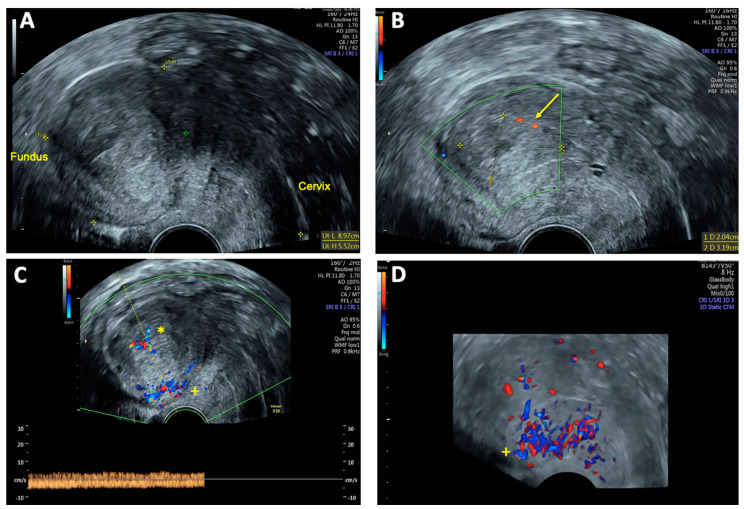
Transvaginal sonography (TVS) 48 h after uterine arteriovenous malformation (AVM) embolization: (**A**) 2D TVS showing an enlarged retroflected uterus measuring 89 × 55 mm post-intervention; (**B**) 2D TVS with color Doppler demonstrating a hypoechoic lesion of 20 × 32 mm with minimal residual perfusion (arrow); (**C**) 2D TVS with color Doppler showing residual venous blood flow in the uterine cavity (*) and intense blood flow localized in the posterior uterine wall (+); (**D**) 3D TVS with color Doppler confirming persistent intense blood flow in the posterior uterine wall (+), with no significant perfusion of the AVM.

Post-interventional TVS performed 48 h after embolization demonstrated a marked reduction in the size of the AVM and the absence of the characteristic Doppler sonographic systolic/diastolic velocity differentiation, indicating successful vascular occlusion (Figure 5). Peridural catheter for analgesia was removed two days post-procedure, and the patient was discharged in stable condition on the same day.

**Figure 6:**
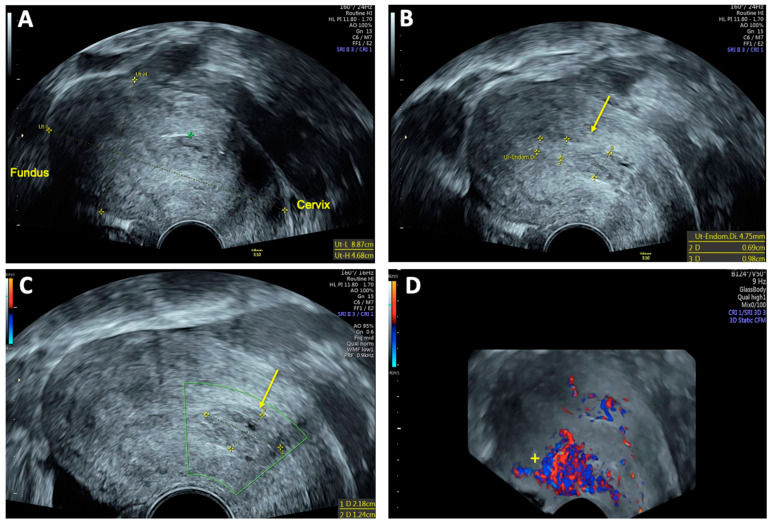
Follow-up transvaginal sonography (TVS) two weeks after uterine arteriovenous malformation (AVM) embolization: (**A**) 2D TVS showing an enlarged retroflected uterus measuring 89 × 47 mm; (**B**) and (**C**) 2D TVS revealing an inhomogeneous vacuolated formation near the cervix measuring 22 × 12 mm (arrow), suspected to be either a former vascular convolute or a blood clot (DD coagulum); (**D**) 3D TVS with color Doppler illustrating high vascularity localized to the posterior uterine wall (+).

Routine follow-up examinations were conducted twice every 2 weeks, followed by monthly assessments after discharge. During the second follow-up visit, the patient reported no other complaints except moderate lower abdominal pain that had begun the previous day. On the day of examination, she experienced her first menstrual period since the curettage, which explained the reported discomfort. TVS showed no residual signs of the previously detected uterine AVM (Figure 6). The endometrial thickness measured 9 mm with no abnormal vascularity. Additionally, an inhomogeneous vacuolated intrauterine formation measuring 22 × 12 mm, located near the cervix, was observed, likely representing either a residual vascular structure without blood flow activity or a blood clot.

**Figure 7:**
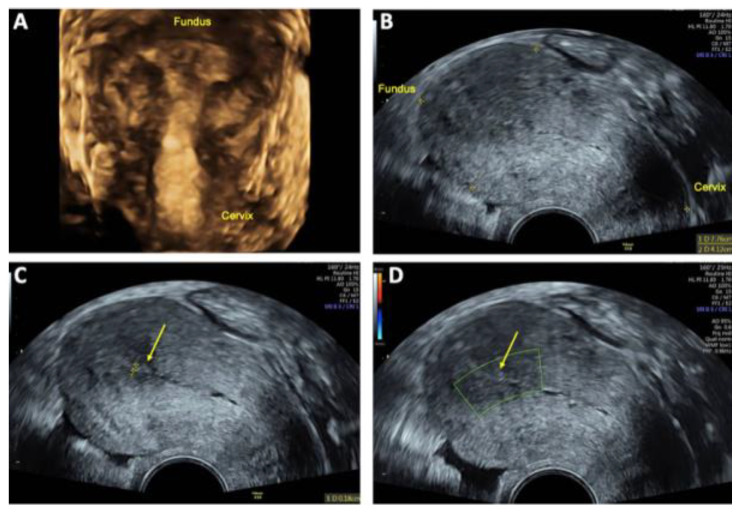
Follow-up transvaginal sonography (TVS) at 6 and 12 weeks after uterine arteriovenous malformation (AVM) embolization: (**A**) 3D TVS at 6 weeks showing no evidence of a lesion within the uterus cavity; (**B**) 2D TVS after 12 weeks demonstrating an almost normal sized retroflected uterus measuring 78 × 41 mm; (**C**) 2D TVS after 12 weeks showing a smooth endometrium with a thickness of 2 mm (arrow); (**D**) 2D TVS after 12 weeks confirming no abnormal perfusion within in the uterine cavity (arrow), indicating successful resolution of the AVM.

At the 12-week follow-up, ultrasound examination showed no residual evidence of the previously detected uterine AVM. The endometrial thickness measured 2 mm with no abnormal vascularity (Figure 7). The patient reported having a regular menstrual cycle without abnormal pain or discomfort, suggesting complete resolution of the AVM and a successful treatment outcome.

**Figure 8:**
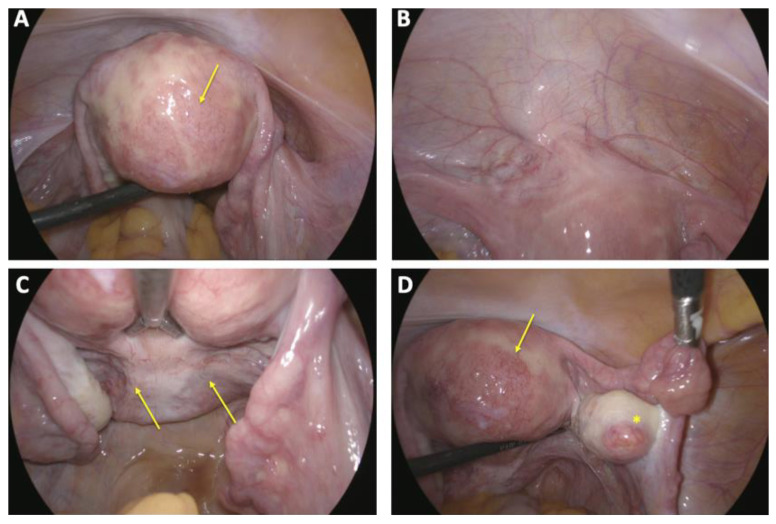
Intraoperative situs laparoscopic finding during laparoscopy with sterilization via bilateral salpingectomy: (**A**) irregularly contoured uterus with scattered and delicate hypervascularization on the posterior uterine wall (arrow); (**B**) bladder peritoneum appearing unremarkable without pathological findings; (**C**) peritoneum of the Pouch of Douglas showing no abnormalities except mild neovascularization (arrow); (**D**) both adnexa demonstrating physiological appearance (*), with a delicate yet notable vascularization on the posterior uterine wall (arrow).

Given the patient’s prior intolerance to hormonal oral contraception and the completion of her family planning, she chose to undergo sterilization via laparoscopic salpingectomy. The procedure was performed without complications during a follow-up visit, three and a half months after embolization. Intraoperative findings showed a uterus with prominent striation and increased vascularization (Figure 8). The patient had an uneventful postoperative course and was discharged a stable condition the following day.

## 3. Discussion

AVM is a rare but serious diagnosis that can lead to significant morbidity, as well as mortality if not diagnosed and treated correctly. It is a known risk following uterine trauma, including curettage, cesarean section, and other surgical interventions on the uterus. In the case study presented here, the patient underwent curettage three times, which makes her a high-risk candidate for the uterine AVM.

This case report highlights that the uterine AVM should be considered not only in women with irregular menstrual bleeding, such as metrorrhagia and menorrhagia, but also in those with unexplained secondary amenorrhea. Early recognition through careful clinical assessment and imaging is essential for timely diagnosis and appropriate management.

The initial diagnosis of uterine AVM is most commonly made using color Doppler ultrasound, which is a rapid, non-invasive, and widely accessible imaging modality. Szpera-Goździewicz et al. described the sonographic appearance of uterine AVMs on the ultrasound picture as “heterogeneous nonspecific spaces in the myometrium, multiple cystic lesions, and tubular anechoic areas concomitant with a normal endometrium”. Additionally, color Doppler findings typically demonstrate “hypervascularity within the lesion, turbulent flow, and multiple tortuous feeding vessels” [8]. Moreover, the PSV and RI are widely recognized in the literature as the most commonly used prognostic indicators for assessing the severity of AVMs and predicting treatment response [10]. In a prospective study by Lee et al., a PSV of <0.52 m/s was associated with a high likelihood of spontaneous resolution, whereas a PSV > 0.70 m/s typically necessitated medical, interventional, or surgical treatment. Another study proposed different cutoff values, suggesting that a PVS < 0.39 m/s is indicative of a spontaneous resolution, while a PVS > 0.83 m/s is more likely to require active intervention [10,11].

To confirm the diagnosis of uterine AVM, DSA remains the gold standard, particularly when transcatheter uterine artery embolization (UAE) is considered as the preferred treatment approach [12]. However, it is important to recognize that DSA is an invasive imaging modality, and its use should be carefully justified. DSA offers detailed visualization of the feeding arteries, facilitating precise embolization planning, while also allowing for the exclusion of differential diagnoses such as GTD or placental tissue retention, both of which may present with similar ultrasound features to AVM [7,13]. In our case, the ß-hCG levels remained mildly elevated three months after curettage, raising suspicion for residual trophoblastic tissue. This highlights the diagnostic challenge of distinguishing uterine AVM from retained placental remnants or GTD. A recent case report described a suspected uterine AVM, which was ultimately diagnosed as placenta increta in a higher gestational stage [14]. Additionally, Timmermann et al. were the first to propose the hypothesis that subinvolution of placental tissue persisting after pregnancy termination or delivery could contribute to the formation of so-called “uterine vascular malformations”—a condition considered distinct from true congenital or acquired AVMs [15]. In this case, the diagnosis of the uterine AVM was confirmed by MRI, and the subsequent indication for UAE following DSA was fully aligned with the recommended diagnostic and therapeutic guidelines. In the present case, the ß-hCG level decreased below the detection threshold before intervention, and this further supported the exclusion of the suspected differential diagnoses, such as GTD or retained placental tissue without diagnostic hysteroscopy.

Transcatheter UAE is widely recognized in the literature as a conservative, first-line treatment for uterine AVMs [8,16], initially described by Forssmann et al. in 1982 [17]. UAE is regarded as a safe and minimally invasive procedure with a manageable risk of complications. Reported adverse effects include low-grade fever, pain, infection, transient buttock and lower limb claudication, perineal skin sloughing, and, in rare cases, uterovaginal and vesico-vaginal fistulae, as well as urinary bladder necrosis [7,12]. Furthermore, several case reports and studies have documented successful post-embolization full-term pregnancies, with reported pregnancy rates up to 77%, indicating that fertility preservation is possible following this procedure [13,18,19]. A systematic review of Peitsidis et al. analyzed 100 cases of acquired uterine AVMs following D&C after termination of pregnancy based on 91 publications. Uterine artery embolization was used in 59%, hysterectomy in 29% and spontaneous resolution in 6%. Follow-up data showed that 17% of patients experienced a recurrence after embolization, and 28% of those who desired future fertility achieved a subsequent pregnancy [20].

Large-sized embolization particles (>500 µm) were selected in our intervention protocol to minimize the risk of ovarian artery occlusion and pulmonary thromboembolism as recommended in the literature [21,22]. In this case, although the patient had completed family planning, she wished to preserve her uterus and opted for the less invasive approach of UAE over surgical intervention. This decision was influenced by the low complication rate and effectiveness of UAE, as well as the shorter post-interventional hospital stay compared to hysterectomy. Given these factors, the UAE was a consensual choice for management.

Recent studies have aimed to distinguish AVMs that carry a high risk of acute hemorrhage and require immediate intervention from those that may be managed conservatively with a watchful waiting approach. This differentiation is primarily guided by Doppler ultrasound parameters, such as PSV and RI, which allow for risk stratification and support individualized treatment decisions [19].

The hysteroscopic approach is also an alternative treatment option for uterine AVM and warrants consideration in selected cases of appropriate small size and no acute hemorrhage. A retrospective cohort study involving 11 participants reported a 100% success rate of AVM elimination, same-day hospital discharge, and four patients subsequently achieving full-term vaginal deliveries following this treatment [11]. However, the study population consisted of patients with active menorrhagia or metrorrhagia, and the average AVM size measured 20 mm, significantly smaller than in our case. Given the greater maximum extension of the AVM in our patient and the absence of future fertility desire, hysteroscopic management was not deemed an optimal approach. Instead, the UAE was selected due to its minimally invasive nature and efficacy in safely managing larger AVMs.

A systematic review analyzing 121 patients across 32 studies evaluated the efficacy of medical management of uterine AVM, reporting an overall success rate of 88% in resolving the vascular abnormality [5]. The study included various monotherapies, such as progestins, GnRH-a, and methotrexate, combined hormonal contraception, uterotonics, and danazol, as well as combinations of these therapies. Among these therapies progestins, GnRH-a and methotrexate were identified as the effective treatment option, with success rates ranging from 82.5% to 90.0%. Furthermore, the lowest complication rates were observed with progestins and GnRH-a, making them the preferred pharmacological choices in cases where medical management is a viable alternative to interventional or surgical treatment. Retrospectively, anti-hormonal therapy with GnRH-a or progestins was not considered a suitable option in our case, due to the patient’s history of depression and the known poor tolerance to hormonal therapies.

The differential diagnosis of GTD and retained placental tissue should always be considered in cases of persistently elevated ß-hCG levels, particularly in patients with a recent history of pregnancy or pregnancy termination, as well as in the presence of hyperperfused areas on Doppler ultrasound.

Although the main symptom of the patient in this case report was amenorrhea, many uterine AVMs are detected due to abnormal uterine bleeding. In this case, adenomyosis should also be an important differential diagnosis, especially in younger women with symptoms such as dysmenorrhea, hypermenorrhea, or secondary infertility. The pathophysiology of adenomyosis remains unclear in detail, but genetic mutation is thought to lead to uncontrolled proliferation of the epithelial component. Investigating adenomyosis as a potential risk factor of uterine AVM could be considered in further research [23]. Furthermore, in case of persistent menometrorrhagia or recurrent abnormal uterine bleeding following curettage or UAE, the possibility of underlying malignant pathologies should be carefully evaluated. This includes endometrial carcinoma, cervical carcinoma, or choriocarcinoma, which must be ruled out, even in young women, to ensure timely diagnosis and appropriate management [9,16].

Given that the patient primarily presented with amenorrhea and slightly elevated levels of β-hCG, it raises the question of whether the amenorrhea was a symptom of the uterine AVM or a result of the curettage or hormonal changes. Since the β-hCG levels continued to decline prior to the procedure and the amenorrhea persisted, causality cannot be established in this context. The effective treatment of the uterine AVM through UAE, which subsequently resulted in the resumption of menstruation, indicates a direct causal relationship. Therefore, it can be concluded that the uterine AVM caused an anatomical change that disrupted the regular menstrual cycle and endometrial development.

**Figure 9:**
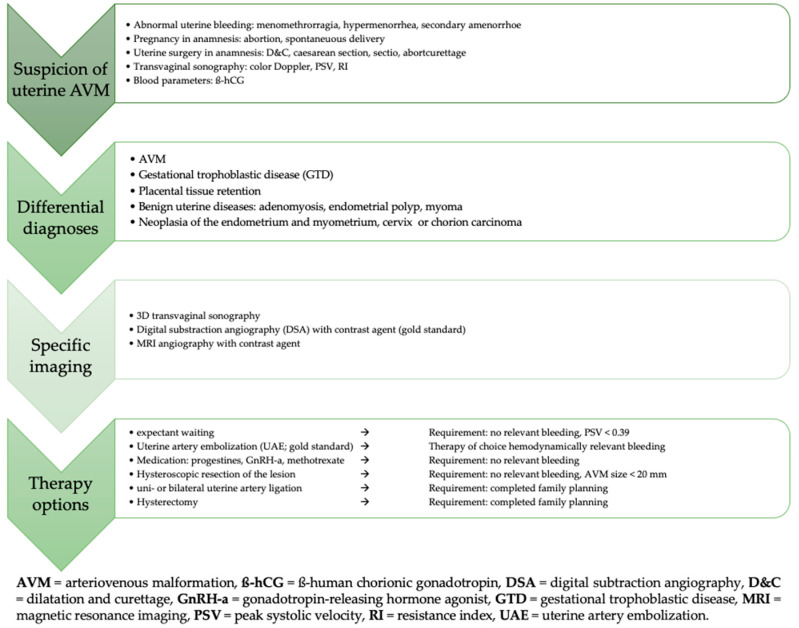
Algorithm summarizing the suspicion, diagnosis, and treatment of uterine AVM.

Regular post-interventional follow-up in our case showed no evidence of recurrence of the uterine AVM and a regular menstrual cycle. However, in the event of symptom recurrence, further therapeutic options, including hysterectomy or additional UAE, particularly of the right uterine artery, should be considered. Given the potential for late recurrence or residual vascular abnormalities, we recommend continuous long-term follow-up to ensure early detection and appropriate management if symptoms reappear. An algorithm for action in the event of suspected uterine AVMs is presented in Figure 9 in a flowchart.

## 4. Conclusions

Uterine AVMs remain a rare but potentially life-threatening condition, if not correctly diagnosed or incorrectly treated, due to the high risk of severe hemorrhage. Therefore, uterine AVMs should always be considered in the differential diagnosis of abnormal uterine bleeding, particularly in women with a medical history of cesarean section, induced abortion, or curettage. Beyond irregular and heavy menstrual bleeding, this case report highlights that the presence of secondary amenorrhea does not exclude the diagnosis of uterine AVM, underscoring the importance of a comprehensive diagnostic approach. This case report demonstrates a stepwise diagnostic process and confirms that UAE is a well-established, standardized, and effective treatment option for uterine AVM, offering successful resolution with a minimal complication rate.

## Data Availability

The datasets analyzed for the current study are available from the corresponding author upon reasonable request.

## Abbreviations

AFP	alpha-fetoprotein
AVM	arteriovenous malformation
ß-hCG	ß-human chorionic gonadotropin
CEA	carcinoembryonic antigen
D&C	dilatation and curettage
DSA	digital subtraction angiography
GnRH-a	gonadotropin-releasing hormone agonist
GTD	gestational trophoblastic diseases
MRI	magnetic resonance imaging
PSV	peak systolic velocity
RI	resistance index
TVS	transvaginal sonography
UAE	uterine artery embolization
